# A predictive model for Gamma Knife intermediate dose spill: R50%_Analytic‐GK_

**DOI:** 10.1002/acm2.14579

**Published:** 2024-11-29

**Authors:** Ivan L. Cordrey, Sare Kucuk, Chester Ramsey, Joseph Bowling, Dharmin D. Desai

**Affiliations:** ^1^ Thompson Cancer Survival Center Cumberland Medical Center Crossville Tennessee USA; ^2^ Department of Nuclear Engineering Complex University of Tennessee Knoxville Tennessee USA; ^3^ Thompson Cancer Survival Center Knoxville Tennessee USA; ^4^ Advanced Oncology Solutions Varian Medical Systems Hixson Tennessee USA

**Keywords:** Gamma Knife, R50%_Analytic_, SRS, surface area, Δr

## Abstract

**Purpose:**

Minimizing intermediate dose spill in stereotactic radiosurgery (SRS) for brain treatment is crucial. Intermediate dose spill correlates with the exposure of normal brain tissue to high doses, which increases the risk of radionecrosis. R50%, defined as the volume of the 50% of prescription isodose cloud/planning target volume, is one metric for intermediate dose spill. A predictive model for R50% in linear accelerator VMAT‐delivered SRS has been developed Desai et al. (2020) and is called R50%_Analytic_. This study extends the R50%_Analytic_ model to Gamma Knife (GK) delivered SRS, resulting in the R50%_Analytic‐GK_ model.

**Methods:**

Phantom calculations were performed on 11 spherical target volumes ranging from 0.001  to 44 cm^3^ to develop the R50%_Analytic‐GK_ model. R50%_Analytic‐GK_ was tested against clinical data from 18 brain metastasis cases with one to 11 targets treated on GK Icon and planned in GammaPlan with lightning dose optimizer. Thirty‐five targets with volumes between 0.011  and 27.4 cm^3^ were analyzed by extracting the R50% achieved clinically (R50%_Clinical_) for comparison to the predicted intermediate dose spill from R50%_Analytic‐GK_.

**Results:**

The predicted R50%_Analytic‐GK_ values generally represent a lower bound for the R50%_Clinical_ values as the model would predict. The Difference, R50%_Clinical_ − R50%_Analytic‐GK_, has a median value of 0.92, which quantifies the lower bound nature of R50%_Analytic‐GK_. The model reflected the character of intermediate dose spill for the clinical cases. A few outliers were likely due to specific planning complexities.

**Conclusion:**

The R50%_Analytic‐GK_ model for intermediate dose spill successfully extends the theoretical framework of R50%_Analytic_ to GK‐delivered SRS. It provides a method to predict the intermediate dose spill for GK Icon treatments. This model can aid in assessing SRS treatment plans by providing a benchmark for the intermediate dose spill for comparison.

## 1 INTRODUCTION

Stereotactic radiosurgery (SRS) and stereotactic radiotherapy (SRT) can be used to deliver high doses of ionizing radiation to cranial targets in one to five fractions with high precision. SRS is frequently used in the treatment of brain targets because of the high tumor control rates and low normal tissue morbidity. Due to the high dose delivered, SRS treatments must be designed to minimize dose to organs at risk (OAR) such as the brainstem, optic chiasm, and cranial nerves. The normal brain is the most vulnerable OAR because it is always in contact with the target volume. Normal brain radionecrosis is one of the most important negative consequences of SRS; thus, limiting intermediate dose spill is critical.

In the conventional Gamma Knife (GK) practice, the intermediate dose spill outside the target is often not a major clinical consideration during treatment planning. The intermediate dose spill is assumed to be as good as can be achieved; if the targets are adequately covered by the prescription isodose cloud and the OAR doses are within published tolerances, the plan is treated. As we see a progression to the treatment of multiple targets in a single session and re‐treatment of new targets in the brain in later courses of SRS, the intermediate dose spill becomes more important to assess and minimize because of the cumulative effects to normal brain tissue.^1^, ^2^, ^3^, ^4^

When assessing intermediate dose spill, the standard metrics involve the volume of the isodose cloud that encompasses 50% of the prescription dose (V_IDC50%_), also known as PIV50% in the literature. Two commonly used intermediate dose spill metrics in SRS are the gradient index (GI) and R50%,

(1) $$\mathrm{GI}\;=\frac{V_{\mathrm{IDC}50}}{V_{\mathrm{IDC}100}}$$

(2) $$R50=\frac{V_{\mathrm{IDC}50}}{V_{\mathrm{PTV}}}$$

where *V*_IDC100%_ is the volume of the isodose cloud that encompasses 100% of the prescription dose and *V*_PTV_ is the planning target volume intended to be treated to the prescription dose (note: R50% is sometimes called mGI).^5^, ^6^, ^7^

In GK convention, the PTV is equivalent to the target volume (i.e., zero margin target for visible disease). For consistency, we use PTV to define the volume intended to be treated to the prescription dose, and we choose R50% for consistency with previously published work in linear accelerator‐based SRS.^8^ R50% also avoids issues previously identified with GI when used as an intermediate dose spill metric.^6^, ^9^

Previous studies have proposed a semi‐empirical model for R50% based on the geometric characteristics of the PTV.^8^ This approach allows for the calculation of a lower threshold value for R50% for a given PTV, called R50%_Analytic_. The R50%_Analytic_ model was developed based on the PTV surface area (SA_PTV_), PTV volume (V_PTV_), PTV effective radius (r_PTV_), and a dose drop‐off parameter (Δr):^8^

(3)

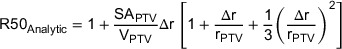

where

(4) $$r_{\mathrm{PTV}}={\left(\frac{3{\;V}_{\mathrm{PTV}}}{4\pi }\right)}^{1/3}$$

**FIGURE 1:**
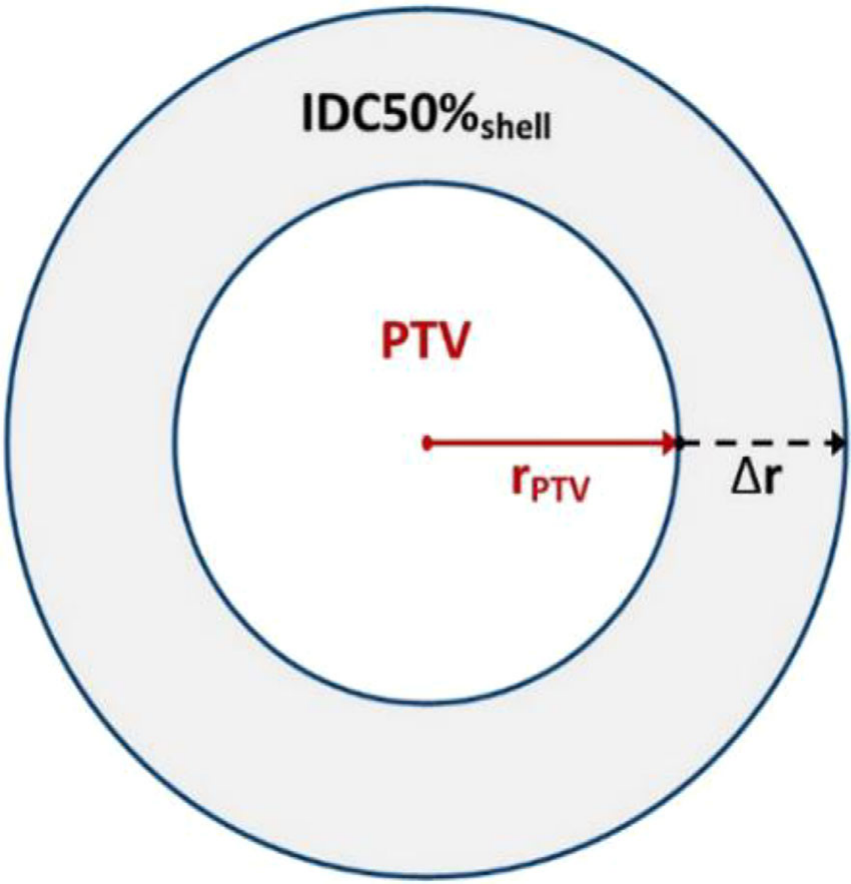
Simple illustration of spherical PTV model. The dose drop‐off parameter (∆r) is the radial distance between the surface of the PTV and the 50% isodose cloud (used by permission^8^).

The dose drop‐off parameter (Δr) is depicted in Figure 1 (used by permission).^8^ ∆r cannot be easily calculated and must be empirically determined from planning studies, as was done in previous works.^8^ Δr is known to be beam energy dependent and machine configuration dependent. Δr should be determined for each energy on each type of treatment machine (e.g., C‐arm linac with a specific MLC, GK, Cyber Knife, etc.), but once determined, Δr can be applied widely for R50%_Analytic_ calculations for any PTV treated on that machine configuration.

The intermediate dose spill of an SRS plan can be benchmarked by comparing the achieved R50% value for a given clinical treatment plan and individual PTV (R50%_Clinical_) with the R50%_Analytic_ calculated for the PTV.

The R50%_Analytic_ model has been previously developed and validated for cranial SRS delivered on C‐arm linacs, specifically TrueBeam (Varian, Palo Alto, California, USA) with VMAT optimization in the Eclipse treatment planning system (Varian, Palo Alto, California, USA).^8^ The R50%_Analytic_ model has not been verified for use with other SRS delivery techniques.

This study extends the R50%_Analytic_ model to the GK Icon and uses clinical data from brain metastases cases to test the model. GammaPlan (Elekta Solutions AB, Stockholm, Sweden) is the dedicated GK planning system. GammaPlan treats a single target at a time and treats multiple targets sequentially by moving each target to the beam convergence point (analogous to a linac isocenter) to deliver the dose to that individual target while ignoring all other targets to be treated in that session of radiation therapy. This process is different from linac single isocenter multiple target treatment delivery, which delivers dose to multiple targets in parallel rather than sequentially. The GammaPlan lightning dose optimizer (LDO) accounts for the dose from treating other targets when designing a multi‐target treatment session plan.

## 2 MATERIALS AND METHODS

### 2.1 Semi‐empirical R50%_Analytic‐GK_ model

The basic model of R50%_Analytic_ is given in Equation 3. To apply this model to GK, one must empirically determine Δr for the GK (Δr_GK_). To determine Δr_GK_, the procedure outlined in the prior R50%_Analytic_ model for C‐arm linacs was employed for the GK Icon.^8^ Thus, a series of test plans were created using the IROC SRS Head Phantom (IROC Houston QA Center, Houston, Texas, USA). Ten spherical PTVs ranging in volume from 0.1 to 44 cm^3^ were positioned in the center of the SRS Head Phantom, and one additional PTV of volume 0.001 cm^3^ was also planned to capture extremely small V_PTV_ behavior.

All plans were created for delivery on the GK Icon, which has 192 sources and a collimator divided into eight sectors. Each sector can have a collimator size of 4 , 8, 16 mm, or can be blocked. The prescribed isodose was adjusted until at least 99% of the PTV received 100% of the prescribed dose: this is described as the D99% volumetric dose coverage. Since the data needed are the volume of the IDC50%, which is for 50% of the prescription dose, the exact prescription dose is irrelevant (the 50% of prescription dose would simply scale with the prescription). But for this phantom study to determine the Δr_GK_, the prescription used was 20 Gy for all 11 spherical PTV phantom plans. The goal of these plans is to determine the minimum distance of dose falloff from the PTV surface to the surface of the 50% of prescription dose isodose cloud (IDC50%) that could be achieved for a spherical target: this defines Δr_GK_.

Inverse treatment planning was performed in GammaPlan version 11.3.1 (Elekta Solutions AB, Stockholm, Sweden) using the LDO.^10^ During the LDO optimization of the 11 phantom plans, no critical tissues were defined. Treatment time was ignored by setting the “Beam On Time Weight” to 0. The dose gradient was maximized by setting the “Low Dose Weight” to 100. Although these GammaPlan settings are not typical for clinical plans because they could generate plans that would exceed the time the patient would be willing to tolerate the treatment procedure, these parameters are only used for these phantom plans that determine Δr_GK_. These settings were selected to provide the plan with the lowest possible intermediate dose spill, regardless of how long the treatment would take to deliver, which results in an idealized minimum intermediate dose spill for the GK Icon.

To confirm that the best possible plan (smallest VIDC50% with the required D99% prescription dose coverage of the PTV) was used to determine Δr_GK_, the 11 spherical PTV phantom cases were replanned by an experienced GK planner. In general, the GammaPlan LDO plans achieved slighltly smaller V_IDC50%_. The best plans (i.e., smallest V_IDC50%_) were chosen between the GammaPlan LDO plans and the experienced planner. Those “best plans” are used to determine the measured Δr_GK_ values.

The smallest collimator (4 mm) is often used as a single shot for very small PTVs whose diameters are less than 4 mm. For these cases, IDC50% remains constant even as the volume of the PTV decreases. Accurately drawing spherical targets with PTVs less than 4 mm diameter is difficult and complicated by the resolution of the image dataset and its inherent voxelation. As such, the very smallest PTV planned in this study (V = 0.001 cm^3^) is a single pixel and was planned as a single 4 mm shot.

Because the PTVs are spherical the radius of the PTV, r_PTV_, can be easily can easily be calculated from the equation of a sphere's volume (as given in Equation 4). The IDC50% for these plans is also spherical owing to the GK's circular collimators and 192 source beam geometry. Thus, with the volume of the IDC50% (V_IDC50%_) determined from the GammaPlan, the radius of the spherical IDC50% can also be determined from Equation 4 by replacing V_PTV_ with V_IDC50%_ and replacing r_PTV_ with r_IDC50%_. Finally, the measured Δr_GK_ can be calculated (with reference to Figure 1):

(5) $${\Delta r}_{\mathrm{GK}}={\;r}_{\mathrm{IDC}50\%}-{\;r}_{\mathrm{PTV}}$$

To mathematically model Δr_GK_ for V_PTV_ between 1 and 44 cm^3^, a power law plus a constant term fit was used. For V_PTV_ smaller than 1 cm^3^ (diameter = 1.24 cm), a different fit was used involving fractional exponent terms plus a constant term. The fit boundary condition that Δr_GK_ (V_PTV_ < 1 cm^3^) equals Δr_GK_ (V_PTV_ > 1 cm^3^) was enforced by adjusting the constant terms in both fit equations. These equations can be used to calculate the Δr_GK_ for the GK Icon over the range of V_PTV_ from 0.001 to 44 cm^3^.

### 2.2 R50%_Analytic‐GK_ validation

Validation of the R50%_Analytic‐GK_ model (Equation 3, with Δr_GK_) was performed using patients treated clinically with single or multiple metastases on a GK Icon. Data was gathered on a total of 46 targets from 18 individual clinical patient plans. The number of targets per plan included nine 1‐PTV plans, five 2‐PTV plans, one 4‐PTV plan, one 5‐PTV plan, one 7‐PTV plan, and one 11‐PTV plan. Because these were clinically treated plans analyzed retrospectively, the prescriptions and GammaPlan LDO optimization settings are typical for clinical GK treatments, and all OAR doses are within accepted clinical ranges as determined by the Gamma Knife Clinical Team attending the case. The prescriptions ranged from 18 to 30 Gy. Again, the data extracted is all referenced as a percentage of the prescription dose (50% of the prescription), thus the actual prescription doses are irrelevant to our analysis of R50%, even though the prescriptions are highly relevant to the individual clinical patients.

The volume of the PTV (V_PTV_) and the volume of the isodose cloud that encompasses 50% of the prescription dose (V_IDC50%_) were extracted from the GammaPlan software, which allows the calculation of R50%_Clinical_ from Equation 2.

There were complications in determining the V_IDC50%_ for some PTVs. Ten PTVs shared an overlapping IDC50% with another PTV and were excluded from the analysis. Further, one PTV was planned as a “single shot” without a contoured PTV on which the plan was based (the PTV was created after the fact). The hastily delineated “single shot” PTV was larger than the 100% isodose line, which is not reasonable, and was also excluded from the analysis. The remaining 35 targets were deemed appropriate for analysis.

The SA_PTV_ can be calculated analytically for spherical targets, but for non‐spherical targets, a Python (Python Software Foundation, Delaware, USA) program was developed to calculate the SA_PTV_ from the DICOM‐RT structure set. This Python script constructs a 3D mesh of a given structure based on the vertices in the RT structure set, and then calculates the surface area of that mesh. The V_PTV_ can be determined directly from the GammaPlan software and then used to analytically calculate the radius of an equivalent sphere (r_PTV_) given by Equation 4. This provides all the necessary inputs to calculate R50%_Analytic‐GK_.

The R50%_Clinical_ is compared to the intermediate dose spill prediction model for the GK, R50%_Analytic‐GK_, calculated from Equation 3 with Δr_GK_ as determined by the phantom study as described in Section 2.1.

## 3 RESULTS

**FIGURE 2:**
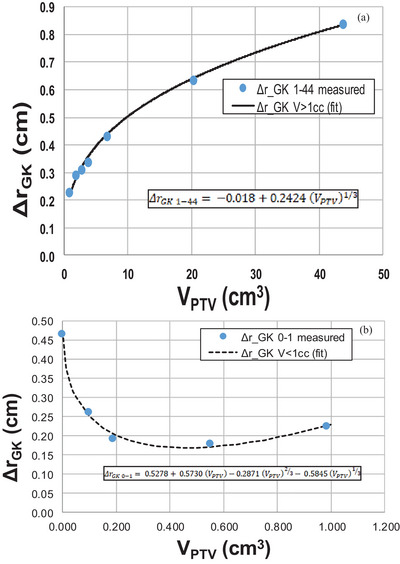
Plots of ∆r_GK_ versus V_PTV_. The measured ∆r_GK_ values (blue circles) are plotted with the fit equations (black curves) in the two relevant PTV volume ranges. When plotted as a function of V_PTV_, the small volume data is highly compressed, and thus, the plots are presented separately: (a) for PTV volumes between 1 and 44 cm^3^ (fit equation [Equation 6] shown as a solid black curve) and (b) for PTV volumes < 1 cm^3^ (fit equation [Equation 7] shown as a dotted black curve).

**FIGURE 3:**
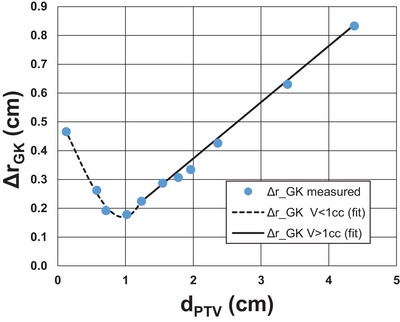
Plot of ∆r_GK_ versus d_PTV_. The measured ∆r_GK_ values (blue circles) and two fit Equations 6 and 7 are plotted as a function of d_PTV_ (black curves). d_PTV_ is the effective diameter of an equivalent volume sphere; thus d_PTV_ is twice the r_PTV_ defined in Equation 4. This is the same data shown in Figure 2. When plotted as a function of d_PTV_, the small volume region is cubically expanded, and the large volume region is cubically compressed. This shows the intersection boundary condition that Δr (V_PTV_ < 1 cm^3^) = Δr (V_PTV_ > 1 cm^3^).

Table 1 and Figures 2 and 3 display the empirically determined Δr values from the phantom study for the 11 spherical PTVs and the fit equations. The Δr_GK_ fit equations are as follows for 1 cm^3^ < V_PTV_ < 44 cm^3^ and V_PTV_ < 1 cm^3^, respectively:

(6) $${\Delta r}_{\mathrm{GK}\;1-44}=-0.018+0.2424{\left(V_{\mathrm{PTV}}\right)}^{1/3}$$

(7) $$\begin{array}{ccc} {\Delta r}_{\mathrm{GK}\;0-1} & = & 0.5278+0.5730\;\left(V_{\mathrm{PTV}}\right)\\ & & -\;0.2871\;{\left(V_{\mathrm{PTV}}\right)}^{2/3}-\;0.5845\;{\left(V_{\mathrm{PTV}}\right)}^{1/3} \end{array}$$

**TABLE 1:** Measured and calculated Δr_GK_ values from the phantom study.

V_PTV_ (cm^3^)	Δr_GK‐measured_ (cm)	Δr_GK 0–1_ (cm)	Δr_GK 1–44_ (cm)
0.001	0.49	0.467	
0.10	0.26	0.252	
0.19	0.19	0.206	
0.55	0.18	0.171	
0.99	0.22	0.227	
1.00	−	0.229	0.224
1.94	0.29		0.284
2.94	0.31		0.329
3.95	0.33		0.365
6.91	0.43		0.444
20.41	0.63		0.644
44.91	0.83		0.844

The Δr_GK‐measured_ values were measured in the study. The ∆r_GK 0–1_ values were calculated from the fit equation for PTV volumes < 1 cm^3^ (Equation 7). The Δr_GK 1–44_ values were calculated from the fit equation for PTV volumes between 1 and 44 cm^3^ (Equation 6).

These fit equations for Δr_GK_ were matched at the boundary V_PTV_ = 1 cm^3^, meaning both equations yield the same result (Δr_GK_ = 0.226 ± 0.003 cm) at V_PTV_ = 1 cm^3^.

**FIGURE 4:**
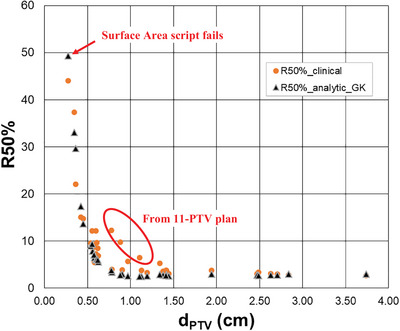
Comparison plot R50%_Clinical_ and R50%_Analytic‐GK_ plotted as functions of d_PTV_. R50%_Clinical_ are from the Gamma Knife clinical cases. d_PTV_ is the effective diameter of an equivalent volume sphere; thus d_PTV_ is twice the r_PTV_ defined in Equation 4. Noted on the graph are the anomalous result for the very smallest PTV (volume = 0.011 cm^3^) and the anomalously high results for R50%_Analytic‐GK_ that occur in a plan with 11 PTVs treated as a single plan in one session.

Figure 4 shows the 35 clinical R50% values plotted with the R50%_Analytic‐GK_ values calculated using Equation 3 and Δr_GK_ (Equation (6), (7)). The data is plotted as a function of d_PTV_, which is the effective diameter of an equivalent volume sphere; thus, d_PTV_ is twice the r_PTV_ defined in Equation 4. This form is used instead of the V_PTV_ because it provides a cubic expansion of the X‐axis for small PTVs (where the majority of the data points lie) and a cubic compression for large PTVs (where data is sparse). This makes it easier to visualize the data and assess the comparison of R50%_Clinical_ and R50%_Analytic‐GK_. The overall character of the R50%_Clinical_ is well modeled by R50%_Analytic‐GK_.

The details of this R50% data are given in Table 2 for each of the 35 PTVs. The table is sorted by Plan Index, which is the Plan number and PTV letter. Thus PTV Index “1A” is plan 1, PTV A. PTV Index “4G” is Plan 4, PTV G (indicating that Plan 4 had at least seven PTVs). The missing letters (4B, 4C, 4E, 4F) are PTVs that were excluded from the analysis for reasons discussed in the Methods section—mainly that the IDC50% of two or more PTVs overlap and cannot be distinguished separately in the final plan. This table includes the surface area of the PTV (SA_PTV_), as determined by the Python script, the clinical prescription dose, and the volumetric dose coverage of the individual PTV (Dn%). Dn% is the percentage of the PTV that receives 100% of the prescribed dose^11^; thus, Dn% = 98.5 means that 98.5% of the PTV's volume receives at minimum the prescription dose. D100% means that the entire PTV (100%) receives full dose, whereas the lowest reported Dn% of 95.9 means that 95.9% of the target volume receives at minimum the prescription dose. The R50%_Clinical_ is calculated from data extracted from GammaPlan by Equation 2. The R50%_Analytic‐GK_ is calculated from Equation 3 using the Δr_GK_ given by the appropriate Equation 6 or 7, depending on the V_PTV_. The last column is the Difference (R50%_Clinical_ − R50%_Analytic‐GK_). The Difference allows the quantitative comparison of the of the two R50% values which are ratios.

**TABLE 2:** Clinical case data.

PTV Index (PlanPTV)	V_PTV_ (cm^3^)	d_PTV_ (cm)	SA_PTV_ (cm^2^)	Prescription dose (Gy)	Dn%	R50%_Clinical_	R50%_Analytic‐GK_	Difference (R50%_clinical_ − R50%_Analytic‐GK_)
1A	0.886	1.19	4.66	20	98.5	3.29	2.53	0.76
2A	0.091	0.56	1.09	22	99.5	8.45	7.87	0.58
2B	0.080	0.53	1.01	22	100.0	9.56	8.93	0.63
2C	0.039	0.42	0.61	22	100.0	15.03	17.37	−2.35
3A	1.378	1.38	6.68	20	98.0	3.61	2.72	0.89
4A	0.113	0.60	1.16	22	99.2	7.21	6.06	1.16
4D	0.480	0.97	3.18	20	99.5	5.66	2.56	3.11
4G	0.011	0.28	0.21	22	100.0	44.00	49.30	−5.30
5A	0.750	1.13	4.13	20	97.5	3.77	2.43	1.33
6A	27.394	3.74	52.34	30	98.6	2.67	2.95	−0.28
7A	3.830	1.94	14.6	18	99.8	3.74	2.95	0.79
7B	0.048	0.45	0.67	22	100.0	14.79	13.61	1.18
8A	0.385	0.90	3.1	20	99.4	3.89	2.97	0.92
9A	10.352	2.70	25.09	27	98.0	2.96	2.76	0.20
10A	0.252	0.78	2.06	20	99.6	3.93	3.40	0.53
11A	9.534	2.63	23.75	27	98.7	3.05	2.76	0.29
11B	0.104	0.58	1.09	27	98.6	5.46	6.47	−1.01
12C	1.486	1.42	7.47	18	100.0	3.77	2.83	0.94
13A	7.917	2.47	20.61	18	98.2	2.92	2.72	0.20
14A	1.576	1.44	6.66	20	98.4	3.07	2.57	0.49
15A	7.895	2.47	20.52	27	99.1	3.09	2.72	0.37
15B	1.256	1.34	6.77	27	98.2	5.27	2.85	2.42
16A	11.971	2.84	31.37	27	98.2	2.75	3.00	−0.25
17C	8.077	2.49	20.78	30	95.9	3.39	2.72	0.67
17D	0.112	0.60	1.14	30	99.8	12.17	6.05	6.12
18A	1.464	1.41	6.77	20	99.7	3.77	2.68	1.09
18B	0.128	0.63	1.29	20	100.0	6.88	5.55	1.32
18C	0.244	0.78	2.24	20	98.5	12.28	3.74	8.54
18D	0.099	0.57	1.11	20	100.0	9.19	7.06	2.13
18E	0.022	0.35	0.45	20	100.0	37.36	33.01	4.35
18F	0.119	0.61	1.29	20	99.8	9.64	6.15	3.49
18G	0.361	0.88	2.6	20	99.9	9.81	2.80	7.01
18H	0.123	0.62	1.32	20	100.0	8.46	5.98	2.47
18I	0.711	1.11	4.07	20	99.8	6.42	2.45	3.97
18J	0.089	0.55	1.28	20	96.0	12.18	9.38	2.80

This is a detailed exposition of the data shown in Figure 4, along with additional plan/PTV information. V_PTV_ is the volume of the PTV, and d_PTV_ is the diameter of an effective sphere of that volume. SA_PTV_ is the surface area of the PTV. Dn% is the percent of PTV covered by the prescription dose. The Difference column is a quantitative comparison of the predicted and clinical values for R50%.

**FIGURE 5:**
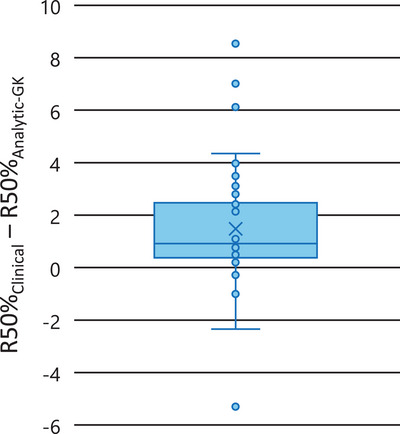
The statistical box plot of the Difference, R50%_Clinical_ − R50%_Analytic‐GK_. This shows the median = 0.92, the quartiles and the whiskers. Note the outlier points: 1 substantially less than the median and 3 substantially greater than the median.

Figure 5 shows the statistical box plot for the Difference, R50%_Clinical_ − R50%_Analytic‐GK_. The median value is 0.92 indicating the R50%_Clinical_ is close to, but generally larger than R50%_Analytic‐GK_. The quartile distribution is skewed positive.

## 4 DISCUSSION

The results shown in Figures 4 and 5 indicate that the predicted R50%_Analytic‐GK_ values manifest the correct character of the R50%_Clinical_. The R50%_Analytic‐GK_ is generally a lower bound for the R50%_Clinical_, which is what one would expect because the Δr_GK_ is derived from the idealized minimum intermediate dose spill for the GK Icon. Not all R50%_Clinical_ values are less than the R50%_Analytic‐GK_, which could be the result of clinical compromises made at the time of planning in some cases. The R50%_Clinical_ values were extracted retrospectively from a collection of clinical cases. This data set is a representative sample of cases that captures a wide range of clinical presentations and unique clinical challenges—it is not a strictly curated dataset.

Figure 4 points out the outliers where the R50%_Clinical_ values fall substantially above or below the R50%_Analytic‐GK_ values. These are the same outlier points seen outside the whiskers on the Figure 5 statistical box plot.

The very smallest volume clinical PTV (V_PTV_ = 0.011 cm^3^, PTV Index 4G) shows an anomaly where the R50%_Clinical_ is substantially smaller than R50%_Analytic‐GK_ by 5.30. This anomaly is explained by a breakdown in the Python surface area script. The reported surface area for this structure is 0.21 cm^2^, while the surface area of a true sphere of the same volume would be 0.24 cm^2^. Since a sphere is the smallest surface area solid object of a given volume, this is a non‐physical result from the Python surface area script and accounts for the anomalous result. Since the Python script uses the vertices of the RT Structure set, and those vertices naturally pixelate any structure, for very small PTVs, the pixilation of the structure surface mesh will have a more substantial impact because there are fewer pixels in the structure. Conceivably, the surface area of the smallest PTV will be impacted more by this pixilation than other larger PTVs in the study. Data point 4G is pointed out by the arrow on Figure 4.

Further, for such small volumes, the counting statistics when determining the IDC50% from the planning system (GammaPlan) can be questionable in that one pixel included in IDC50% or excluded from IDC50% could be an appreciable difference in the reported volume. Thus, this very smallest PTV cannot be weighted heavily in our assessment of the validity of R50%_Analytic‐GK_, and yet, it does show that R50%_Analytic‐GK_ correctly represents the general character of the R50% intermediate dose spill metric in such small clinical targets.

Further investigation of the outlier PTVs with R50%_Clinical_ noticeably larger than the R50%_Analytic‐GK_ reveals that three of the PTVs (circled on the plot of Figure 4) are from a plan with 11‐PTVs. This is likely the result of an “over‐spray” effect, a low‐dose wash that results from the unintended dose to a PTV, while the other PTVs in the plan are being treated.

In general, this data confirms the R50%_Analytic‐GK_ model and ∆r_GK_ are applicable to the GK Icon and GammaPlan. Since the R50%_Analytic_ model is a theoretical geometric result, this is not surprising. The ∆r_GK_ determined for the very limited phantom calculations of 11 spherical single PTV phantom plans yields a predictive model of intermediate dose spill for the GK Icon.

The major limitation of applying the full R50%_Analytic‐GK_ plan assessment in GK is that the surface area of the PTV is not reported within the GammaPlan software. The SA_PTV_ determination requires porting DICOM data to an external Python script. Of course, a surface area calculation could be incorporated into GammaPlan by the vendor, which would make the calculation of R50%_Analytic‐GK_ simple. A number of journal articles have demonstrated the correlation of dose spill with target surface area in SRS.^8^, ^12^, ^13^, ^14^, ^15^, 14 As such, it could be very useful for all planning software to incorporate a reported surface area for target volumes in SRS planning, and we encourage GammaPlan to include such surface area reporting in future software versions. In the absence of an easily obtainable surface area for targets in GammaPlan, or the writing and validation of an surface area script (like our Python surface area script), the user could approximate the surface area of the target with the surface area of an equivalent volume sphere. For many smaller targets, the PTV is often nearly spherical, so this could be a very reasonable approximation. For larger targets, this approximation may be substantially less accurate depending on the shape of the PTV. Using a spherical approximation will capture much of the character of the R50% curves depicted in Figure 4. But, since a sphere has the smallest possible surface area of a given volume solid object, one would predicted from Equation 3, that the R50%_Analytic‐GK_ calculated with this spherical approximation would be less than or equal to the R50%_Analytic‐GK_ one would calculate using the actual PTV surface area.

The GK Icon is not the only Cobalt‐60 source device available for radiation therapy. It is interesting to ask if this predictive model for intermediate dose spill, R50%_Analytic‐GK_, can be applied to other Cobalt‐60 source SRS therapy machines. The fundamental structure of R50%_Analytic_ is geometric, so for any highly conformal treatment delivery system, the fundamental equation of R50%_Analytic_ (Equation 3) should be a valid approximation (although this is not directly verified by this work). In principle, the empirically determined Δr parameter should be directly determined for each unique machine configuration. This would require only the calculation of ten to 11 phantom plans with spherical PTVs (as described in the Methods 2A). It is known from unpublished investigations in C‐arm linear accelerator VMAT‐delivered SRS that the parameter Δr is dependent on the photon beam energy with higher energy photons yielding a larger value of Δr for all target volumes, and that the Δr is not strongly dependent on the exact MLC employed on the C‐arm linear accelerators (except for very small PTVs). This implies that the Δr_GK_ determined for a GK Icon could have some validity for other Cobalt‐60 treatment machines, but we also know that Δr_GK_ is dependent on machine configuration details. This is evident in the Δr_GK_ plot for V_PTV_ < 1 cm^3^ (Figure 2b), which is depicted in Figure 3 for d_PTV_ < 12.4 mm (the dash line) where we observe a dramatic change in the character of the Δr_GK_ curve relative to the character of the Δr_GK_ curve for larger PTVs. As stated in the Materials and Methods, for very small PTVs, the smallest collimator available on the GK Icon (4 mm) is used and the target is treated as a single shot meaning the IDC50% remains constant for PTVs smaller than 1 cm^3^. This is a machine‐specific characteristic owing to the nature of the smallest collimator available on the GK Icon. The behavior of Δr for very small PTVs could be quite different for a Cobalt‐60 beam with a different beam shaping device like an MLC. This is a potential avenue for further investigation.

One unique aspect of GammaPlan is that the calculation dose grid is not universal to the image data set but is specifically tied to each target and of limited extent. Thus, the dose calculation grid for PTV1 covers only the local region of PTV1 but not the entire image data set. Similarly, the dose calculation grid for PTV2 covers only the local region of target two, which may or may not overlap with the dose grid of PTV1, but, likewise, does not cover the entire image dataset used to plan the treatment. At the conclusion of planning, the individual dose grids are summed to give a picture of the entire planned session of radiation therapy. One does have the option to add an additional dose grid that covers both PTVs and the entire merged IDC50% from which one could extract the volume of the entire overlapping merged IDC50%, but this is an additional step that historically has not added clinical value in the absence of a good metric to assess the merged IDC50% or R50%. Thus, it is often impractical in GammaPlan to extract the combined V_IDC50%_ for a cluster of targets that are close enough together that their individual IDC50% volumes overlap. Further, there is no tool to convert an isodose surface to a 3D contour, which would facilitate the extraction of the V_IDC50%_.

Extracting data on the combined IDC50% of PTVs in close proximity is typically not done within GammaPlan due to the limitations of the software tools. In GK clinical practice, one examines the dose distribution of each PTV individually, and if the individual conformalities appear adequate, it is assumed the composite plan's conformality is adequate as long as the dose constraints on critical organs, such as optic pathway and brainstem, are below established guidelines. In the case of PTVs in close proximity whose IDC50% overlap, the GK clinical practice disregards the GI as reported by GammaPlan. However, now that the R50%_Analytic‐GK_ model has been validated, one could use the “Fair Value Estimate” (FVE) for R50% as described by Desai and Cordrey to apportion the combined/overlapping IDC50%.^12^, ^13^ Utilizing the FVE method would achieve an R50% for such cases that could be compared to the R50%_Analytic_.

The accurate modeling of intermediate dose spill is crucial for optimizing SRS treatments, as it helps in minimizing the dose to surrounding normal tissues, especially proximal normal brain, while ensuring effective tumor dose. The R50%_Analytic‐GK_ model can serve as a tool in assessing GK treatments, providing a reliable method to predict and thus control intermediate dose spill. By validating the model with clinical data, we have demonstrated its applicability. As stated in the Introduction, controlling intermediate dose spill is increasingly important in an era of SRS re‐treatment and multiple PTVs treated in a single session. This validation also underscores the importance of using patient‐specific data to refine and validate treatment planning models to ensure both accuracy and clinical relevancy.

This method of plan assessment using R50%_Analytic‐GK_ should be more important for highly non‐spherical targets, such as arteriovenous malformations and pituitary adenomas, where the expected intermediate dose spill is more difficult to assess.^10^ Because R50%_Analytic‐GK_ directly accounts for the target surface area, this could provide important plan assessment information in highly non‐spherical PTV cases such as arteriovenous malformations and pituitary adenomas.

## 5 CONCLUSION

The development and validation of the R50%_Analytic‐GK_ model for the GK Icon has demonstrated its robustness and applicability in predicting intermediate dose spill for a range of PTV volumes. By incorporating geometric characteristics of the target volume such as surface area, volume, and effective radius, along with an empirically determined dose drop‐off parameter (Δr_GK_), the model provides a reliable method for assessing SRS treatment plans.

The R50%_Analytic‐GK_ model can offer value in clinical practice, particularly for complex and highly non‐spherical targets where dose distribution assessments are challenging. Its ability to provide accurate intermediate dose spill predictions could help in developing the optimal treatment plan.

## AUTHOR CONTRIBUTIONS

**Ivan L. Cordrey**: Conception and design of study; data analysis and interpretation; manuscript drafting. **Sare Kucuk**: Data acquisition; initial data analysis. **Chester Ramsey**: Design of study; data acquisition; data analysis and interpretation. **Joseph Bowling**: creation of original clinical plans used in the study; data interpretation. **Dharmin D. Desai**: Conception of study; data interpretation. All authors participated in manuscript revisions.

## CONFLICT OF INTEREST STATEMENT

The authors declare no conflicts of interest.

## Data Availability

The data that support the findings of this study are available from the corresponding author upon reasonable request.
